# Chemical Glucosylation of Labile Natural Products Using a (2‐Nitrophenyl)acetyl‐Protected Glucosyl Acetimidate Donor

**DOI:** 10.1002/ejoc.201800260

**Published:** 2018-04-26

**Authors:** Julia Weber, Markus Schwarz, Andrea Schiefer, Christian Hametner, Georg Häubl, Johannes Fröhlich, Hannes Mikula

**Affiliations:** ^1^ Institute of Applied Synthetic Chemistry Vienna University of Technology (TU Wien) Getreidemarkt 9 1060 Vienna Austria; ^2^ Romer Labs Technopark 1 3430 Tulln/Donau Austria

**Keywords:** Glycosylation, Protecting groups, Diastereoselectivity, Natural products, Neighboring‐group effects

## Abstract

The synthesis of (2‐nitrophenyl)acetyl (NPAc)‐protected glucosyl donors is described that were designed for the neighboring‐group assisted glucosylation of base‐labile natural products also being sensitive to hydrogenolysis. Glycosylation conditions were optimized using a trichloroacetimidate glucosyl donor, and cyclohexylmethanol and (+)‐menthol as model acceptors. The approach was then extended to a one‐pot procedure for the synthesis of 1,2‐*trans*‐glycosides. This method was finally applied for improved synthesis of the masked mycotoxin T2‐*O*‐β,d‐glucoside.

## Introduction

Glycosides are widespread in nature, often found as secondary metabolites formed in plants or mammals during xenobiotic metabolism.^1^, ^2^ A variety of glycosylated bioactive natural products is known with the sugar part playing a pivotal role regarding biological activity and recognition of cellular targets.^3^, ^4^ Considering the limited availability of glycosides from biological sources and the need of significant amounts of these materials for biological, medicinal and pharmacological studies, the chemical synthesis of glycoconjugates is of high interest.^5^ However, the glycosylation of complex natural products still remains a challenging task as there is no general procedure known so far for the diastereoselective synthesis of glycoconjugates.^6^ The use of peracetylated glycosyl donors represents the most commonly applied method exploiting the neighboring participating effect of the acetyl group for the formation of 1,2‐*trans* glycosides.^7^, ^8^ However, in case of a base‐labile natural product (e.g. compounds containing ester groups), these donors fail as soon as it comes to deprotection of the acetyl groups on the sugar moiety. To circumvent this problem, several strategies have been developed and reported including benzyl‐protected donors with a neighboring participating group at position 2 such as AZMB,^9^ MSc,^10^ or picolinyl.^11^ These donors can efficiently be used for the diastereoselective glycosylation of base‐sensitive acceptor molecules. However, these donors are inapplicable for the glycosylation of natural products that are not stable under hydrogenolytic conditions (e.g. compounds containing carbon–carbon double or triple bonds). There are only very few glycosyl donor systems known so far that might be applied for the glycosylation of natural products being sensitive to both basic conditions and hydrogenolysis. For the synthesis of (–)‐cassiode a fully PMB‐protected sulfoxide glucosyl donor was used, which surprisingly even showed enhanced diastereoselectivity toward β‐glycosylation.^12^ Heuckendorff et al.^13^ used a similar strategy (PMB‐protected thioglycosyl donor) and showed that the selectivity of β‐glycosylation is strongly dependent on the acceptor. A fully TBDMS‐protected sulfoxide glucosyl donor was applied for the synthesis of glycopeptides^14^ and a fully TIPS‐protected thioethyl glucosyl donor was described by Okada et al.,^15^ with both showing good β‐selectivities in glycosylation reactions with cyclohexylmethanol due to the restricted twist‐boat conformation. However, in case of more complex alcohols as acceptors a decreased diastereoselectivity was observed when using this glucosyl donor.^16^ Hence, a reliable strategy for the glycosylation of complex and labile natural products is still missing. To the best of our knowledge, no donor system has been reported so far making use of neighboring group participation for diastereoselective 1,2‐*trans* glycosylation of base‐labile natural products containing at least one carbon–carbon double bond.

**Figure 1:**
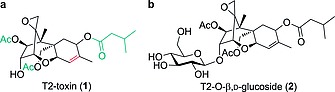
Chemical structure of (a) T2‐toxin and (b) its glycosylated metabolite T2‐O‐β,d‐glucoside.

In the course of ongoing research in the field of phase II metabolites of mycotoxins, we have become interested in the development of glucosyl donors that can be used for the glucosylation of T2‐toxin (**1**, Figure 1a), a compound that contains ester groups and a carbon–carbon double bond. T2‐toxin is a potent inhibitor of the eukaryotic protein synthesis and represents a contaminant of considerable concern to human and animal health.^17^ Masked T2‐toxins, especially glycosides, can emerge after metabolizing in plants and fail to be recognized in conventional analyses.^2^ As hydrolysis to the parent toxin can occur during digestion,^18^, ^19^ these masked toxins present a risk considering the underestimation of the total mycotoxin content in routine screenings. For the development of routine analyses including masked mycotoxins^20^ such as T2‐glucoside (**2**, Figure 1b) and further studies regarding toxicity and structure elucidation, there is an urgent need for sufficient amounts of reference materials.

**Figure 2:**
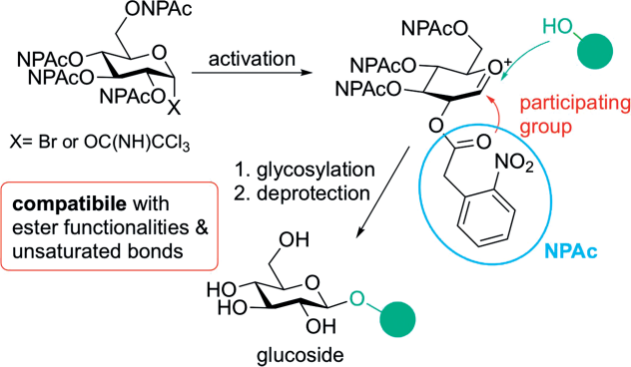
NPAc‐protected glucosyl donors for glycosylation of labile acceptors.

As described previously, glycosylation of T2‐toxin was achieved under thioglycosylation conditions using a TIPS‐protected thioethyl glucosyl donor as developed by Okada et al.,^15^ leading to an isomeric 5:1 mixture of β‐ and α‐glucoside. The aim to improve this reaction considering the reduction of elaborate purification steps and increased versatility of the glycosylation reaction led us to the development of a new glucosyl donor system using (2‐nitrophenyl)acetyl (NPAc) as neighboring participating group (Figure 2). NPAc has been developed by Daragics et al.^21^ for the protection of hydroxy groups and seemed to us to be the perfect choice as its cleavage can be carried out under very mild reductive conditions. Moreover, NPAc can be introduced using commercially available nitrophenylacetic acid and it was already shown that this protective group provides a participation effect in glycosylation reactions leading to formation of 1,2‐*trans* glycosides.^21^

Herein, we present the synthesis and application of a glucosyl donor that is suitable for the glycosylation of base‐labile natural products that are not compatible with hydrogenolytic removal of protecting groups.

## Results and Discussion

**Scheme 1:**
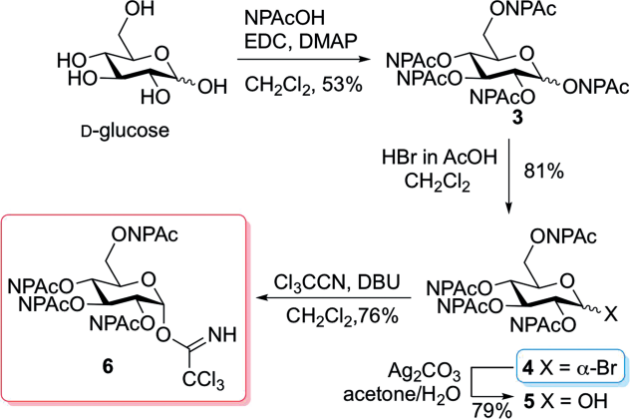
Synthesis of 2,3,4,6‐tetra‐O‐NPAc protected glucosyl donors **4** (bromosugar) and **6** (trichloroacetimidate).

Starting from d‐glucose 1,2,3,4,6‐NPAc‐protected glucose **3** was prepared applying a slightly modified protocol as described by Daragics et al.^21^ using coupling of (2‐nitrophenyl)acetic acid to the OH‐groups of the sugar mediated by 1‐ethyl‐3‐(3‐dimethylaminopropyl)carbodiimide (EDC) and catalytic amounts of 4‐dimethylaminopyridine (DMAP). **3** was treated with HBr in acetic acid^5^ to obtain bromosugar **4** that can be used for Königs–Knorr glycosylation. Hydrolysis of the anomeric center with silver carbonate in acetone/water^22^ yielded the 1‐hydroxy sugar **5** that was subsequently reacted with trichloroacetonitrile and 1,8‐diazabicyclo[5,4,0]‐undec‐7‐ene (DBU)^23^ to obtain the trichloroacetimidate donor **6** for Schmidt glycosylation (Scheme 1). Notably, first attempts to introduce the imidoyl group by reacting **5** with trichloroacetonitrile and potassium carbonate^24^ failed, while using DBU as a base led to full conversion after 16 h affording **6** in 76 %. In contrast to the report of Jacquinet et al. describing modification of NPAc groups in the presence of DBU and Cl_3_CCN,^25^ we have not observed any side reaction.

To investigate glycosylation applying the NPAc‐protected donors **4** and **6**, cyclohexylmethanol was used as model acceptor with a primary OH‐group. Selected data of reaction optimization and screening is shown in Table 1. Königs Knorr glycosylation using bromosugar **4** as donor activated by various silver salts resulted in no conversion after 24 h (for examples see Entry 1 and Entry 2), while Lewis‐acid mediated glycosylation applying donor **6** and BF_3_ **·**Et_2_O as promoter led to formation of product **7**, but only in low yield (Entry 3). Further screening was carried out considering various Lewis acids, number of equivalents of reactants and promoter, solvent, etc., to obtain an improved protocol using TMSOTf as activator for the trichloroacetimidate donor **6** to obtain **7** in 80 % yield (Entry 4). Encouraged by the results we tested the applicability of the optimized procedure towards secondary alcohols using (+)‐menthol as a model acceptor, and were able to obtain compound **8** in 64 % (Entry 5).

**Table 1:** Glycosylation of cyclohexylmethanol (CyMeOH) and (+)‐menthol using glycosyl donors **4** and **6**

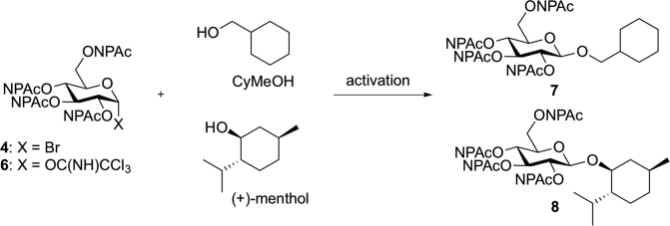					
Entry	Donor	Acceptor	Activation	Solvent	Yield [%]^a^
	[equiv.]	[equiv.]	[equiv.]		(product)
1	**4** (1.0)	CyMeOH (1.2)	Ag_2_O (1.5)	MeCN	0 (**7**)
2	**4** (1.0)	CyMeOH (1.2)	Ag_2_CO_3_ (1.5)	CH_2_Cl_2_	0 (**7**)
3	**6** (1.0)	CyMeOH (1.2)	BF_3_ **·**Et_2_O (0.1)	CH_2_Cl_2_	5 (**7**)
4	**6** (1.2)	CyMeOH (1.0)	TMSOTf (0.1)	CH_2_Cl_2_	80 (**7**, β/α = 6:1)
5	**6** (1.2)	(+)‐Menthol (1.0)	TMSOTf (0.1)	CH_2_Cl_2_	64 (**8**, β/α = 8:1)

a Non‐isolated yield as determined by HPLC (using standard addition for quantification).

Unexpectedly, even though glucosyl donor **6** is equipped with a neighboring participating group at O2, all Schmidt glycosylation reactions led to a mixture of 1,2‐*trans* and 1,2‐*cis* glycosides. However, the formation of β‐glucosides was still favored with a β/α ratio of up to 8:1. We hypothesize that the conformation of the sugar and/or the intermediate oxocarbenium species (after activation) is influenced due to steric hindrance of the four NPAc groups thus preventing more efficient participation.

**Scheme 2:**
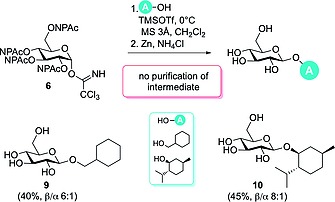
“One‐pot” procedure for glycosylation using the NPAc‐protected glucosyl trichloroacetimidate donor **6**.

Cleavage of NPAc‐groups was finally achieved under reductive conditions with zinc and ammonium chloride^21^ supported by ultrasonic irradiation, and thus under reaction conditions compatible with ester functionalities and unsaturated carbon–carbon bonds. As the NPAc‐protected glycosides **7** and **8** were obtained in a complex product mixture, purification of these intermediates turned out to be very laborious. To avoid this time‐consuming step, we developed a “one‐pot” procedure (only including filtration and concentration of the crude glycosylation reaction mixture) using cyclohexylmethanol and (+)‐menthol as acceptors, leading to the deprotected glucosides **9** and **10** in overall yields of 40 and 45 %, respectively (Scheme 2).

**Scheme 3:**
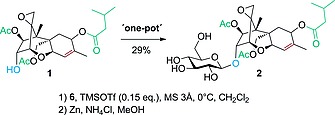
Glycosylation of T2‐toxin (**1**) and subsequent deprotection of the crude intermediate to obtain T2‐O‐β,d‐glucoside (**2**).

Applying this method, we were able to synthesize T2‐O‐β,d‐glucoside starting from T2‐toxin. Following the optimized Schmidt‐glycosylation procedure the protected intermediate could be obtained in a crude mixture of isomers in a β/α ratio of 8:1 (as determined by HPLC). Subsequent deprotection (after filtration and concentration of the crude reaction mixture) under reductive conditions and purification by reversed‐phase column chromatography afforded T2‐O‐β,d‐glucoside (**2**) in an overall yield of 29 % (Scheme 3).

## Conclusions

In summary, we were able to develop a new glycosyl donor system that can be applied for the synthesis of glycosides of base‐labile acceptors that furthermore contain unsaturated carbon–carbon bonds, and thus cannot be glycosylated using previously described methods that make use of participating protecting groups that are cleaved by hydrogenolysis. Reaction conditions for the glycosylation step were optimized using model acceptors (primary and secondary alcohols) and finally simplified to obtain a “one‐pot” procedure to avoid laborious purification of the protected intermediates. Applying this optimized procedure, we could facilitate the synthesis of T2‐O‐β,d‐glucoside and achieved an enhancement of the selectivity of the glycosylation reaction towards the β‐isomer of the intermediate. Due to the ease and low‐cost preparation of the glucosyl donor using commercially available reagents, we are convinced that this new donor system represents a valuable tool for complex glycosylation reactions.

## Experimental Section

All reactions were performed under an argon atmosphere. Anhydrous solvents (dichloromethane, tetrahydrofuran, methanol and diethyl ether) were dried using a PURESOLV facility of it‐innovative technology. Molecular sieves (3 Å) were activated under vacuum at 200 °C. Reaction progress was monitored by LC‐ESI‐MS/MS performed with an HCT ion trap mass spectrometer (Bruker, Germany) in full scan mode. Chromatographic separation was done using a 1200 series HPLC system (Agilent Technologies, Germany) and a Luna RP‐C18 column (3.0 × 150 mm, 3 µm particle size, Phenomenex, Germany). Preparative column chromatography was performed on silica gel 60 (Merck, 40–63 µm) using a Büchi Sepacore^TM^ Flash System. Preparative HPLC separation was done with a Büchi Reveleris Prep system using a Luna Prep C18(2), 10 µm, 250 × 10 mm column (Phenomenex). NMR spectra were recorded with a Bruker Avance IIIHD 600Mhz spectrometer equipped with a Prodigy BBO cryoprobe or a Bruker Avance DRX‐400 MHz spectrometer at 20 °C. Data were recorded and evaluated using TOPSPIN 3.5 (Bruker Biospin). All chemical shifts are given in ppm relative to tetramethylsilane. The calibration was done using residual solvent signals. T2‐toxin was provided by Romer Labs (Tulln) and all other chemicals were purchased from ABCR (Germany) or Sigma–Aldrich (Austria/Germany). HRMS analysis was carried out by analyzing acetonitrile solutions (concentration: 10 ppm) on an Agilent 6230 AJS ESI–TOF mass spectrometer after chromatographic separation on an Agilent 1100/1200 HPLC (Agilent Technologies, Waldbronn, Germany).

**1,2,3,4,6‐Penta‐*O*‐(2‐nitrophenyl)acetyl‐d‐glucopyranoside (3):** To a solution of glucose (1.0 g, 5.6 mmol, 1 equiv.) and 2‐nitrophenylacetic acid (6.1 g, 33.6 mmol, 6 equiv.) in dry dichloromethane (50 mL) (4‐dimethylamino)pyridine (1.1 g, 5.6 mmol, 1 equiv.) and EDC (6.4 g, 5.6 mmol, 6 equiv.) were added. The reaction mixture was stirred at room temperature for 16 h, washed with 1 n HCl (2 × 50 mL) and saturated NaHCO_3_ solution (2 × 50 mL), dried with Na_2_SO_4_, filtered and concentrated. The crude product was purified by flash chromatography (CH_2_Cl_2_/EtOAc, gradient elution 100:1 to 20:1) to obtain the title compound as a white foam (3.0 g, 53 %). According to NMR analysis, a 4:1 (α:β) mixture of anomers was obtained. ^1^H NMR (400 MHz, CDCl_3_): **α**‐**(4)**: *δ* = 8.20–8.11 (m, 4 H), 8.09 (dd, *J* = 8.2, 1.2 Hz, 1 H), 7.70–7.57 (m, 5 H), 7.56–7.46 (m, 8 H), 7.43 (td, *J* = 7.8, 1.3, 1 H), 7.34 (dd, *J* = 7.6, 1.3 Hz, 1 H), 6.37 (d, *J* = 3.5 Hz, 1 H), 5.59 (t, *J* = 9.9 Hz, 1 H), 5.14 (t, *J* = 9.9 Hz, 1 H), 5.08 (dd, *J* = 10.1, 3.9 Hz, 1 H), 4.39 (dd, *J* = 12.3, 1.4 Hz, 1 H), 4.32–4.09 (m, 9 H), 4.08–3.93 (m, 3 H); **β‐(4)**: *δ* = 8.20–8.11 (m, 4 H), 8.09 (dd, *J* = 8.2, 1.2 Hz, 1 H), 7.70–7.57 (m, 5 H), 7.56–7.46 (m, 8 H), 7.43 (td, *J* = 7.8, 1.3, 1 H), 7.34 (dd, *J* = 7.6, 1.3 Hz, 1 H), 5.79 (d, *J* = 8.2 Hz, 1 H), 5.43 (t, *J* = 9.7 Hz, 1 H), 5.22 (dd, *J* = 9.7, 8.2 Hz, 1 H), 5.14 (t, *J* = 9.7 Hz, 1 H), 4.36 (dd, *J* = 12.5, 4.6 Hz, 1 H), 4.32–4.09 (m, 9 H), 4.08–3.93 (m, 3 H) ppm. ^13^C NMR (100 MHz, CDCl_3_): **α‐(4)**: *δ* = 169.81 (s, 2 C), 169.62 (s, 1 C), 169.29 (s, 1 C), 168.50 (s, 1 C), 148.75 (s, 1 C), 148.57 (s, 1 C), 148.56 (s, 1 C), 148.52 (s, 1 C), 148.49 (s, 1 C), 134.42 (d, 1 C), 134.32 (d, 1 C), 134.25 (d, 1 C), 134.15 (d, 1 C), 134.12 (d, 3 C), 133.91 (d, 1 C), 133.76 (d, 1 C), 133.73 (d, 1 C), 130.11 (s, 1 C), 129.88 (s, 1 C), 129.80 (s, 1 C), 129.59 (s, 1 C), 129.52 (s, 1 C), 129.07 (d, 1 C), 128.89 (d, 1 C), 128.87 (d, 1 C), 128.77 (d, 1 C), 128.65 (d, 1 C), 125.54 (d, 1 C), 125.35 (d, 2 C), 125.31 (d, 1 C), 125.25 (d, 1 C), 89.29 (d, 1 C), 70.18 (d, 1 C), 70.10 (d, 1 C), 69.84 (d, 1 C), 68.11 (d, 1 C), 62.48 (t, 1 C), 39.84 (t, 1 C), 39.74 (t, 1 C), 39.69 (t, 1 C), 39.55 (t, 1 C), 39.50 (t, 1 C) ppm. **β‐(4)**: *δ* = 169.69 (s, 1C), 169.66 (s, 1C), 169.24 (s, 1C), 169.18 (s, 1C), 168.86 (s, 1C), 148.56 (s, 1C), 148.52 (s, 1C), 148.49 (s, 1C), 148.46 (s, 1C), 148.42 (s, 1C), 134.56 (d, 1C), 134.45 (d, 1C), 134.25 (d, 1C), 134.15 (d, 1C), 134.12 (d, 3C), 133.82 (d, 1C), 133.76 (d, 1C), 133.73 (d, 1C), 130.06 (s, 1C), 129.88 (s, 1C), 129.83 (s, 1C), 129.55 (s, 1C), 129.46 (s, 1C), 129.00 (d, 1C), 128.89 (d, 1C), 128.87 (d, 1C), 128.80 (d, 1C), 128.61 (d, 1C), 125.42 (d, 1C), 125.35 (d, 2C), 125.31 (d, 1C), 125.25 (d, 1C), 91.97 (d, 1C), 72.86 (d, 1C), 72.37 (d, 1C), 70.51 (d, 1C), 68.32 (d, 1C), 62.15 (t, 1C), 39.62 (t, 1C), 39.55 (t, 1C), 33.91 (t, 1C), 25.67 (t, 1C), 24.99 (t, 1C) ppm. HRMS calcd. for C_46_H_37_N_5_NaO_21_^+^ [M + Na]^+^ 1018.1873, found 1018.1875.

**1‐Bromo‐1‐deoxy‐2,3,4,6‐tetra‐*O*‐(2‐nitrophenyl)acetyl‐α,d‐glucopyranose (4):** To a solution of compound **3** (4.0 g, 4.02 mmol, 1 equiv.) in dry dichloromethane (3 mL) hydrogen bromide in acetic acid [30 % (wt), 5 mL] was slowly added at 0 °C. The reaction was warmed to room temperature and stirred for 4 h and then quenched by the addition of ice water (30 mL). The mixture was diluted with CH_2_Cl_2_ and washed with saturated aqueous NaHCO_3_ solution. The organic layer was dried with Na_2_SO_4_ and the solvents were removed under reduced pressure to give the desired product **4** as a yellow foam (2.9 g, 81 %). ^1^H NMR (600 MHz, CDCl_3_): *δ* = 8.19–8.14 (m, 3 H), 8.09 (dd, *J* = 8.2, 1.2 Hz, 1 H), 7.67–7.57 (m, 4 H), 7.53–7.47 (m, 5 H), 7.46–7.42 (m, 2 H), 7.31 (dd, *J* = 7.7, 0.9 Hz, 1 H), 6.51 (d, *J* = 4.1 Hz, 1 H), 5.66 (t, *J* = 9.7 Hz, 1 H), 5.21 (t, *J* = 9.8 Hz, 1 H), 4.96 (dd, *J* = 9.8, 3.9 Hz, 1 H), 4.38 dd (*J* = 13.1, 4.2 Hz, 1 H), 4.33–4.29 (m, 2 H), 4.27 (d, *J* = 17.0 Hz, 1 H), 4.22–4.14 (m, 3 H), 4.08 (d, *J* = 17.0 Hz, 1 H), 4.03 (d, *J* = 17.0 Hz, 1 H), 3.97 (d, *J* = 5.3 Hz, 1 H), 3.95 (d, *J* = 5.6 Hz, 1 H) ppm. ^13^C NMR (150 MHz, CDCl_3_): *δ* = 146.35 (s, 1 C), 146.20 (s, 1 C), 146.08 (s, 1 C), 146.82 (s, 1 C), 125.40 (s, 1 C), 125.15 (s, 3 C), 111.07 (d, 1 C), 110.88 (d, 2 C), 110.84 (d, 2 C), 110.80 (d, 1 C), 110.39 (d, 1 C), 110.38 (d, 1 C), 106.73 (s, 1 C), 106.28 (s, 1 C), 106.17 (s, 1 C), 106.13 (s, 1 C), 105.72 (d, 1 C), 105.64 (d, 1 C), 105.54 (d, 1 C), 105.40 (d, 1 C), 102.16 (d, 1 C), 102.07 (d, 1 C), 102.03 (d, 1 C), 102.02 (d, 1 C), 63.26 (d, 1 C), 49.02 (d, 2 C), 47.56 (d, 1 C), 46.99 (d, 1 C), 38.28 (t, 1 C), 16.45 (t, 3 C), 16.24 (t, 1 C) ppm. ESI‐MS calcd. for C_38_H_32_BrN_4_O_17_^+^ [M + H]^+^ 895.1, found 895.1.

**2,3,4,6‐Tetra‐*O*‐(2‐nitrophenyl)acetyl‐d‐glucopyranose (5):** To a solution of compound **4** (1.45 g, 1.67 mmol, 1 equiv.) in acetone (9 mL) water (30 µL) and Ag_2_CO_3_ (0.52 g, 1.84 mmol, 1.1 equiv.) were added. After stirring at room temperature for 20 h in the dark, the reaction mixture was filtered through Celite and the filtrate was concentrated under reduced pressure. The residue was dissolved in DCM and filtered through a short pad of silica gel (elution with EtOAc/hexanes, 1:1) to obtain a 4:1 (α:β) mixture of anomers of **5** (1.1 g, 79 %). ^1^H NMR (400 MHz, CDCl_3_): **α‐(6)**: *δ* = 8.21–8.08 (m, 4 H), 7.68–7.54 (m, 5 H), 7.53–7.41 (m, 6 H), 7.36–7.30 (m, 1 H), 5.65 (t, *J* = 9.9 Hz, 1 H), 5.43 (d, *J* = 3.5 Hz, 1 H), 5.03 (t, *J* = 9.8 Hz, 1 H), 4.94 (dd, *J* = 10.1, 3.5 Hz, 1 H), 4.35–4.26 (m, 2 H), 4.25–4.18 (m, 2 H), 4.18–4.11 (m, 3 H), 4.10–4.00 (m, 3 H), 3.95 (d, *J* = 17.6 Hz, 1 H), 3.37 (br. s, 1 H) ppm. **β‐(6)**: *δ* = 8.21–8.08 (m, 4 H), 7.68–7.54 (m, 5 H), 7.53–7.41 (m, 6 H), 7.36–7.30 (m, 1 H), 5.39 (t, *J* = 9.9 Hz, 1 H), 5.08 (t, *J* = 9.9 Hz, 1 H), 4.97 (dd, *J* = 9.8, 8.2 Hz, 1 H), 4.77 (d, *J* = 7.8 Hz, 1 H), 4.35–4.26 (m, 2 H), 4.25–4.18 (m, 2 H), 4.18–4.11 (m, 3 H), 4.10–4.00 (m, 3 H), 3.95 (d, *J* = 17.6 Hz, 1 H), 3.37 (br. s, 1 H) ppm. ^13^C NMR (100 MHz, CDCl_3_): **α‐(6)**: *δ* = 169.88 (s, 1 C), 169.67 (s, 1 C), 169.57 (s, 1 C), 169.43 (s, 1 C), 148.52 (s, 4 C), 134.37 (d, 1 C), 134.18 (d, 1 C), 134.10 (d, 4 C), 133.95 (d, 1 C), 133.78 (d, 1 C), 130.23 (s, 1 C), 129.98 (s, 2 C), 129.67 (s, 1 C), 128.89 (d, 1 C), 128.86 (d, 1 C), 128.75 (d, 2 C), 125.46 (d, 1 C), 125.44 (d, 1 C), 125.35 (d, 1 C), 125.28 (d, 1 C), 90.14 (d, 1 C), 71.78 (d, 1 C), 70.07 (d, 1 C), 69.05 (d, 1 C), 67.24 (d, 1 C), 62.91 (t, 1 C), 39.95 (t, 1 C), 39.82 (t, 2 C), 39.77 (t, 1 C). **β‐(6)**: 170.09 (s, 1 C), 169.80 (s, 1 C), 169.57 (s, 1 C), 169.35 (s, 1 C), 148.61 (s, 1 C), 148.52 (s, 2 C), 148.49 (s, 1 C), 134.47 (d, 1 C), 134.23 (d, 1 C), 134.10 (d, 4 C), 133.95 (d, 1 C), 133.78 (d, 1 C), 130.12 (s, 1 C), 129.92 (s, 1 C), 129.72 (s, 1 C), 129.63 (s, 1 C), 128.94 (d, 1 C), 128.86 (d, 1 C), 128.79 (d, 1 C), 128.75 (d, 1 C), 125.46 (d, 1 C), 125.44 (d, 1 C), 125.31 (d, 1 C), 125.28 (d, 1 C), 95.49 (d, 1 C), 73.77 (d, 1 C), 72.21 (d, 1 C), 72.19 (d, 1 C), 68.87 (d, 1 C), 62.59 (t, 1 C), 39.90 (t, 1 C), 39.82 (t, 1 C), 39.71 (t, 1 C), 39.64 (t, 1 C) ppm. HRMS calcd. for C_38_H_32_N_4_NaO_18_^+^ [M + Na]^+^ 855.1603, found 855.1604. ppm.

**2,3,4,6‐Tetra‐*O*‐(2‐nitrophenyl)acetyl‐α,d‐glucopyranosyl Trichloroacetimidate (6):** To a solution of compound **5** (1.2 g, 1.4 mmol, 1 equiv.) in dry CH_2_Cl_2_, trichloroacetonitrile (43 µL, 4.3 mmol, 3 equiv.) was slowly added at 0 °C, followed by DBU (21 µL, 0.14 mmol, 0.1 equiv.). The reaction mixture was slowly warmed to room temperature and stirring was continued for 16 h. The solution was concentrated under vacuum and the crude product was purified by flash chromatography (EtOAc in hexanes, gradient elution) to obtain the title compound as a lightly yellow foam (1.07 g, 76 %). ^1^H NMR (600 MHz, CDCl_3_): *δ* = 8.70 (s, 1 H), 8.17 (dd, *J* = 8.3, 1.1 Hz, 1 H), 8.15 (dd, *J* = 8.0, 1.2 Hz, 1 H), 8.12 (dd, *J* = 8.2, 1.2 Hz, 1 H), 8.01 (dd, *J* = 8.2, 1.3 Hz, 1 H), 7.69–7.56 (m, 4 H), 7.54–7.41 (m, 7 H), 7.31 (dd, *J* = 7.6, 1.2 Hz, 1 H), 6.52 (d, *J* = 3.8 Hz, 1 H), 5.71 (t, *J* = 9.8 Hz, 1 H), 5.25 (t, *J* = 9.9 Hz, 1 H), 5.22 (dd, *J* = 10.1, 3.7 Hz, 1 H), 4.35 (dd, *J* = 12.6, 3.8 Hz, 1 H), 4.31 (dd, *J* = 12.6, 1.7 Hz, 1 H), 4.24 (ddd; *J* = 10.5, 3.9, 2.1 Hz, 1 H), 4.22 (d, *J* = 6.5 Hz, 1 H), 4.20–4.14 (m, 3 H), 4.09 (d, *J* = 16.9 Hz, 1 H), 3.99 (d, *J* = 14.9 Hz, 1 H), 3.96 (d, *J* = 14.1 Hz, 1 H), 3.91 (d, *J* = 17.3 Hz, 1 H) ppm. ^13^C NMR (150 MHz, CDCl_3_): *δ* = 169.76 (s, 1 C), 169.69 (s, 1 C), 169.50 (s, 1 C), 168.24 (s, 1 C), 160.61 (s, 1 C), 148.76 (s, 1 C), 148.56 (s, 1 C), 148.48 (s, 1 C), 148.44 (s, 1 C), 143.43 (d, 1 C), 134.21 (d, 2 C), 134.10 (d, 1 C), 134.08 (d, 1 C), 133.69 (d, 2 C), 133.65 (d, 1 C), 130.14 (s, 1 C), 129.64 (s, 1 C), 129.57 (s, 1 C), 129.47 (s, 1 C), 128.97 (d, 1 C), 128.90 (d, 1 C), 128.83 (d, 1 C), 128.66 (d, 1 C), 125.44 (d, 1 C), 125.35 (d, 1 C), 125.31 (d, 2 C), 92.96 (d, 1 C), 90.80 (s, 1 C), 70.33 (d, 1 C), 70.27 (d, 1 C), 69.93 (d, 1 C), 67.80 (d, 1 C), 62.10 (t, 1 C), 39.76 (t, 1 C), 39.69 (t, 1 C), 39.52 (t, 1 C), 39.49 (t, 1 C) ppm. HRMS calcd. for C_40_H_32_Cl_3_N_5_NaO_18_^+^ [M + Na]^+^ 998.0700, found 998.0702.

**General Procedure A: Königs‐Knorr Glycosylation:** To a solution of alcohol (0.01 mmol, 1 equiv.) and glucosyl donor **4** (80 mg, 0.013 mmol, 1.2 equiv.) in dry CH_2_Cl_2_ or MeCN (1 mL) was added molecular sieves (3 Å, 0.1 g/mL). After stirring at room temperature for 1 h, promoter (0.017 mmol, 1.5 equiv.) was added and the reaction mixture was stirred in the dark for 24 h. A sample was taken and analyzed by HPLC. Standard addition was used for quantification.

**General Procedure B: Schmidt Glycosylation:** To a solution of alcohol (0.07 mmol, 1 equiv.) and glucosyl donor **6** (80 mg, 0.08 mmol, 1.2 equiv.) in dry CH_2_Cl_2_ (1 mL) molecular sieves (3 Å, 0.1 g/mL) was added, and the reaction mixture was stirred at room temp. for 1 h. After cooling the reaction mixture to 0 °C, TMSOTf (1.6 µL, 9 µmol, 0.15 equiv.) was added. The reaction mixture was stirred for 2 h, and then quenched by the addition of Et_3_N (0.15 equiv.). The reaction mixture was filtered through Celite and concentrated. The crude product was purified by flash chromatography (EtOAc in hexanes, gradient elution) to obtain the desired product.

**1‐Methylcyclohexyl‐2,3,4,6‐tetra‐*O*‐(2‐nitrophenyl)acetyl‐β,d‐glucopyranoside (7):** General procedure A; starting from cyclohexyl methanol (12.3 mg, 0.11 mmol) and glucosyl donor **6** (127 mg, 0.13 mmol) **7** was obtained as a white solid (32 mg, 49 %). ^1^H NMR (400 MHz, CDCl_3_): *δ* = 8.21–8.04 (m, 4 H), 7.68–7.42 (m, 10 H), 7.41–7.29 (m, 2 H), 5.33 (t, *J* = 9.6 Hz, 1 H), 5.07 (t, *J* = 9.8 Hz, 1 H), 4.99 (dd, *J* = 10.0, 7.7 Hz, 1 H), 4.46 (d, *J* = 8.2 Hz, 1 H), 4.38 (dd, *J* = 12.3, 1.8 Hz, 1 H), 4.33–4.16 (m, 3 H), 4.14–4.08 (m, 2 H), 4.07–3.81 (m, 4 H), 3.70 (ddd, *J* = 9.9, 5.5, 1.8 Hz, 1 H), 3.65 (dd, *J* = 9.4, 5.8 Hz, 1 H), 3.26 (dd, *J* = 9.6, 6.8 Hz, 1 H), 1.80–1.60 (m, 4 H), 1.31–1.15 (m, 4 H), 1.00–0.78 (m, 3 H) ppm. ^13^C NMR (100 MHz, CDCl_3_): *δ* = 169.81 (s, 1 C), 169.73 (s, 1 C), 169.34 (s, 1 C), 168.77 (s, 1 C), 148.84 (s, 1 C), 148.79 (s, 1 C), 148.65 (s, 1 C), 148.52 (s, 1 C), 134.49 (d, 1 C), 134.21 (d, 1 C), 134.12 (d, 1 C), 133.99 (d, 1 C), 133.85 (d, 1 C), 133.80 (d, 1 C), 133.71 (d, 1 C), 133.68 (d, 1 C), 130.16 (s, 1 C), 129.82 (s, 1 C), 129.71 (s, 1 C), 129.65 (s, 1 C), 128.85 (d, 1 C), 128.80 (d, 1 C), 128.75 (d, 1 C), 128.64 (d, 1 C), 125.41 (d, 1 C), 125.31 (d, 2 C), 125.26 (d, 1 C), 101.05 (d, 1 C), 75.76 (t, 1 C), 72.66 (d, 1 C), 72.13 (d, 1 C), 72.00 (d, 1 C), 68.95 (d, 1 C), 62.88 (t, 1 C), 39.76 (t, 2 C), 39.62 (t, 2 C), 37.86 (d, 1 C), 29.88 (t, 1 C), 29.70 (t, 1 C), 26.69 (t, 1 C), 25.96 (t, 1 C), 25.91 (t, 1 C) ppm. HRMS calcd. for C_45_H_44_N_4_NaO_18_^+^ [M + Na]^+^ 951.2543, found 951.2536.

**1‐(+)‐Menthyl‐2,3,4,6‐tetra‐*O*‐(2‐nitrophenyl)acetyl‐β,d‐glucopyranoside (8):** General procedure A; starting from (+)‐menthol (10.7 mg, 0.06 mmol) and glucosyl donor **6** (80 mg, 0.08 mmol) **8** was obtained as a slightly yellow solid (18 mg, 31 %). ^1^H NMR (400 MHz, CDCl_3_): *δ* = 8.18–8.05 (m, 4 H), 7.65–7.40 (m, 10 H), 7.35 (dd, *J* = 7.8, 1.6 Hz, 1 H), 7.32 (dd, *J* = 7.4, 1.2 Hz, 1 H), 5.36 (t, *J* = 9.6 Hz, 1 H), 5.07 (t, *J* = 9.7 Hz, 1 H), 5.03 (dd, *J* = 9.8, 7.8 Hz, 1 H), 4.61 (d, *J* = 8.2 Hz, 1 H), 4.44 (dd, *J* = 12.1, 1.9 Hz, 1 H), 4.27 (d, *J* = 17.6 Hz, 1 H), 4.22–4.03 (m, 6 H), 3.92 (d, *J* = 17.2 Hz, 1 H), 3.76 (d, *J* = 17.6 Hz, 1 H), 3.74 (ddd, *J* = 10.1, 5.9, 2.0 Hz, 1 H), 3.42 (td, *J* = 10.7, 4.3 Hz, 1 H), 2.25–2.07 (m, 2 H), 1.73–1.50 (m, 3 H), 1.41–1.23 (m, 3 H), 1.12–1.01 (m, 1 H), 0.95 (d, *J* = 7.0 Hz, 3 H), 0.88 (d, *J* = 6.6 Hz, 3 H) 0.83 (d, *J* = 7.0 Hz, 3 H) ppm. ^13^C NMR (100 MHz, CDCl_3_): *δ* = 169.89 (s, 1 C), 169.73 (s, 1 C), 169.42 (s, 1 C), 168.74 (s, 1 C), 148.87 (s, 1 C), 148.83 (s, 1 C), 148.68 (s, 1 C), 148.57 (s, 1 C), 134.49 (d, 1 C), 134.22 (d, 1 C), 134.05 (d, 1 C), 133.92 (d, 1 C), 133.86 (d, 1 C), 133.68 (d, 1 C), 133.67 (d, 1 C), 133.61 (d, 1 C), 130.15 (s, 1 C), 129.80 (s, 1 C), 129.70 (s, 1 C), 129.59 (s, 1 C), 128.94 (d, 1 C), 128.74 (d, 1 C), 128.69 (d, 1 C), 128.66 (d, 1 C), 125.51 (d, 1 C), 125.31 (d, 1 C), 125.26 (d, 1 C), 125.19 (d, 1 C), 101.82 (d, 1 C), 82.31 (d, 1 C), 72.88 (d, 1 C), 72.50 (d, 1 C), 71.82 (d, 1 C), 69.13 (d, 1 C), 63.13 (t, 1 C), 48.27 (d, 1 C), 43.07 (t, 1 C), 39.66 (t, 1 C), 39.65 (t, 1 C), 39.56 (t, 1 C), 39.47 (t, 1 C), 34.23 (t, 1 C), 31.77 (d, 1 C), 25.08 (d, 1 C), 22.86 (t, 1 C), 22.40 (q, 1 C), 21.48 (q, 1 C), 16.23 (q, 1 C) ppm. HRMS calcd. for C_48_H_50_N_4_NaO_18_^+^ [M + Na]^+^ 993.3012, found 993.3012.

**General Procedure C: “One‐Pot” Glycosylation/Deprotection:** To a solution of alcohol (1 equiv.) and glycosyl donor **6** (1.5 equiv.) in dry CH_2_Cl_2_ (25 mL/mmol) was added molecular sieves (3 Å, 0.1 g/mL), and the reaction mixture was stirred at room temp. for 1 h. After cooling to 0 °C, TMSOTf (0.15 equiv.) was added and reaction mixture was stirred at 0 °C. After 2 h, the reaction was quenched by the addition of Et_3_N (0.15 equiv.), filtered through Celite and concentrated. The crude product mixture was dissolved in MeOH (17 mL/mmol), and zinc dust (20 equiv.) and ammonium chloride (12 equiv.) were added at room temp. The reaction was treated with ultrasonic irradiation for 2–4 h at 25 °C, then filtered through Celite and concentrated under vacuum. Preparative HPLC (RP‐C18, MeCN in H_2_O, gradient elution) afforded the desired glycoside.

**Methylcyclohexyl‐β,d‐glucopyranoside (9):** General procedure C; starting from cyclohexyl methanol (14.6 mg, 0.13 mmol) and glucosyl donor **6** (150 mg, 0.15 mmol) **9** was obtained as a colorless solid (14 mg, 40 %). According to NMR analysis, a 6:1 (β/α) mixture of anomers was obtained. ^1^H NMR (400 MHz, CD_3_OD): *δ* = 4.24 (d, *J* = 7.4 Hz, 1 H), 3.87 (dd, *J* = 12.2, 1.9 Hz, 1 H), 3.73 (dd, *J* = 9.4, 6.6 Hz, 1 H), 3.70–3.65 (m, 1 H), 3.39–3.23 (m, 4 H), 3.18 (dd, *J* = 8.8, 7.9 Hz, 1 H), 1.91–1.78 (m, 2 H), 1.77–1.58 (m, 4 H), 1.38–1.15 (m, 3 H), 1.07–0.90 (m, 2 H) ppm. ^13^C NMR (100 MHz, CD_3_OD): *δ* = 104.59 (d, 1 C), 78.14 (d, 1 C), 77.89 (d, 1 C), 76.53 (t, 1 C), 75.17 (d, 1 C), 71.68 (d, 1 C), 62.77 (t, 1 C), 39.35 (d, 1 C), 31.06 (t, 1 C), 31.03 (t, 1 C), 27.73 (t, 1 C), 26.99 (t, 1 C), 26.97 (t, 1 C) ppm. HRMS calcd. for C_14_H_25_O_8_^–^ [M + COOH]^–^ 321.1555, found 321.1558.

**(+)‐Menthyl‐β,d‐glucopyranoside (10):** General procedure C; starting from (+)‐menthol (10.7 mg, 0.07 mmol) and glucosyl donor **6** (80 mg, 0.08 mmol) **10** was obtained as a white solid (10 mg, 45 %). According to NMR analysis, a 8:1 (β/α) mixture of anomers was obtained. ^1^H NMR (400 MHz, CD_3_OD): *δ* = 4.32 (d, *J* = 7.8 Hz, 1 H), 3.85 (dd, *J* = 11.9, 2.2 Hz, 1 H), 3.67 (dd, *J* = 11.7, 5.1 Hz, 1 H), 3.43 (td, *J* = 10.7, 4.3 Hz, 1 H), 3.39–3.32 (m, 1 H), 3.30–3.21 (m, 2 H), 3.16 (t, *J* = 8.2 Hz, 1 H), 2.45 (m, *J* = 13.9, 6.9, 2.4 Hz, 1 H), 2.33–2.22 (m, 1 H), 1.73–1.60 (m, 2 H), 1.48–1.32 (m, 1 H), 1.31–1.22 (m, 1 H), 1.09–0.93 (m, 2 H), 0.91 (d, *J* = 6.0 Hz, 3 H), 0.90 (d, *J* = 7.6 Hz, 3 H), 0.88–0.83 (m, 1 H), 0.80 (d, *J* = 7.0 Hz, 3 H) ppm. ^13^C NMR (100 MHz, CD_3_OD): *δ* = 105.69 (d, 1 C), 82.44 (d, 1 C), 78.19 (d, 1 C), 77.74 (d, 1 C), 75.59 (d, 1 C), 71.68 (d, 1 C), 62.81 (t, 1 C), 50.23 (d, 1 C), 44.74 (t, 1 C), 35.60 (t, 1 C), 32.94 (d, 1 C), 25.71 (d, 1 C), 24.00 (t, 1 C), 22.72 (q, 1 C), 21.63 (q, 1 C), 16.31 (q, 1 C) ppm. HRMS calcd. for C_16_H_29_O_6_^+^ [M – H]^–^ 317.1970, found 317.1971.

**T2‐O‐β,d‐glucoside (2):** General procedure C; starting from T2‐toxin (30 mg, 0.06 mmol) and glucosyl donor **6** (94 mg, 0.096 mmol) T2‐β,d‐glucoside **(2)** was obtained as a white solid (11 mg, 29 %). ^1^H NMR (600 MHz, CD_3_OH): *δ* = 5.98 (d, *J* = 3.2 Hz, 1 H), 5.78 (dt, *J* = 6.0; 1.0 Hz, 1 H), 5.33 (d, *J* = 5.6 Hz, 1 H), 4.48 (dd, *J* = 5.0, 3.0 Hz, 1 H), 4.45 (d, *J* = 7.9 Hz, 1 H), 4.39 (d, *J* = 6.7 Hz, 1 H), 4.38 (d, *J* = 12.9 Hz, 1 H), 4.09 (d, *J* = 12.7 Hz, 1 H), 3.84 (dd, *J* = 12.0, 2.0 Hz, 1 H), 3.72 (d, *J* = 5.0 Hz, 1 H), 3.65 (dd, *J* = 12.1, 5.7 Hz, 1 H), 3.35 (t, *J* = 9.0 Hz, 1 H), 3.29 (t, *J* = 8.8 Hz, 1 H), 3.25 (dd, *J* = 8.9, 7.5 Hz, 1 H), 3.21 (ddd, *J* = 9.5, 5.7, 2.5 Hz, 1 H), 3.04 (d, *J* = 3.8 Hz, 1 H), 2.87 (d, *J* = 3.8 Hz, 1 H), 2.38 (dd, *J* = 15.2, 5.9 Hz, 1 H), 2.16 (dd, *J* = 7.0, 2.3 Hz, 2 H), 2.09 (s, 3 H), 2.07 (s, 3 H), 2.06–2.03 (m, 1 H), 1.94 (d, *J* = 15.3 Hz, 1 H), 1.75 (s, 3 H), 0.97 (d, *J* = 4.5 Hz, 3 H), 0.96 (d, *J* = 4.4 Hz, 3 H), 0.74 (s, 3 H) ppm. ^13^C NMR (150 MHz, CD_3_OH): *δ* = 173.97 (1 C), 172.27 (1 C), 172.19 (1 C), 137.37 (1 C), 125.07 (1 C), 103.78 (1 C), 83.84 (1 C), 81.17 (1 C), 80.48 (1 C), 78.34 (1 C), 78.06 (1 C), 74.79 (1 C), 71.43 (1 C), 69.32 (1 C), 68.49 (1 C), 65.38 (1 C), 65.26 (1 C), 62.59 (1 C), 50.07 (1 C), 47.86 (1 C), 44.50 (1 C), 44.32 (1 C), 28.76 (1 C), 26.94 (1 C), 22.77 (1 C), 22.71 (1 C), 21.21 (1 C), 20.80 (1 C), 20.44 (1 C), 7.04 (1 C) ppm. HRMS calcd. for C_31_H_45_O_16_^–^ [M + COOH]^–^ 673.2713, found 673.2716.

## Supporting information

Supporting Information
