# Supplementary material for: Chemical Glucosylation of Labile Natural Products Using a (2‐Nitrophenyl)acetyl‐Protected Glucosyl Acetimidate Donor
Source: European J Org Chem. 2018 Apr 26;2018(20-21):2701–6. doi: 10.1002/ejoc.201800260 (PMC6001546; doi:10.1002/ejoc.201800260)

Eur. J. Org. Chem. • ISSN 1099–0690

https://doi.org/10.1002/ejoc.201800260

SUPPORTING INFORMATION

Title: Chemical Glucosylation of Labile Natural Products Using a (2-Nitrophenyl)acetyl-Protected Glucosyl Acetimidate Donor

Author(s): Julia Weber, Markus Schwarz, Andrea Schiefer, Christian Hametner, Georg Häubl, Johannes Fröhlich, Hannes Mikula*

NMR spectra

a. 1,2,3,4,6-Penta-*O*-(2-nitrophenyl)acetyl-β,D-glucopyranoside (3)

^{1}H NMR (d-CDCl$_3$, 400 MHz)

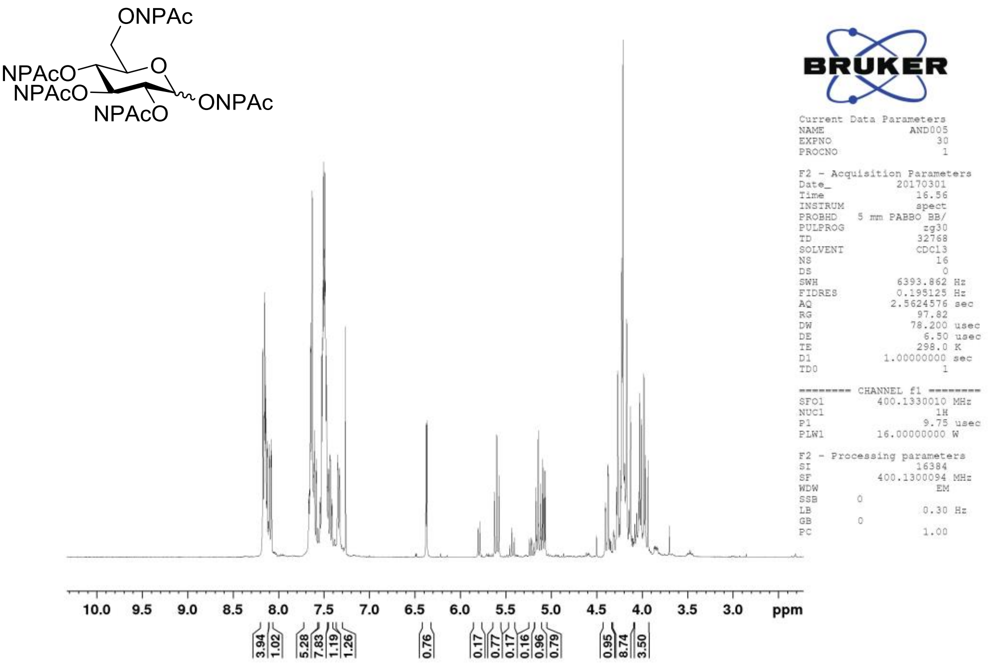

^{13}C NMR (d-CDCl$_3$, 100 MHz)

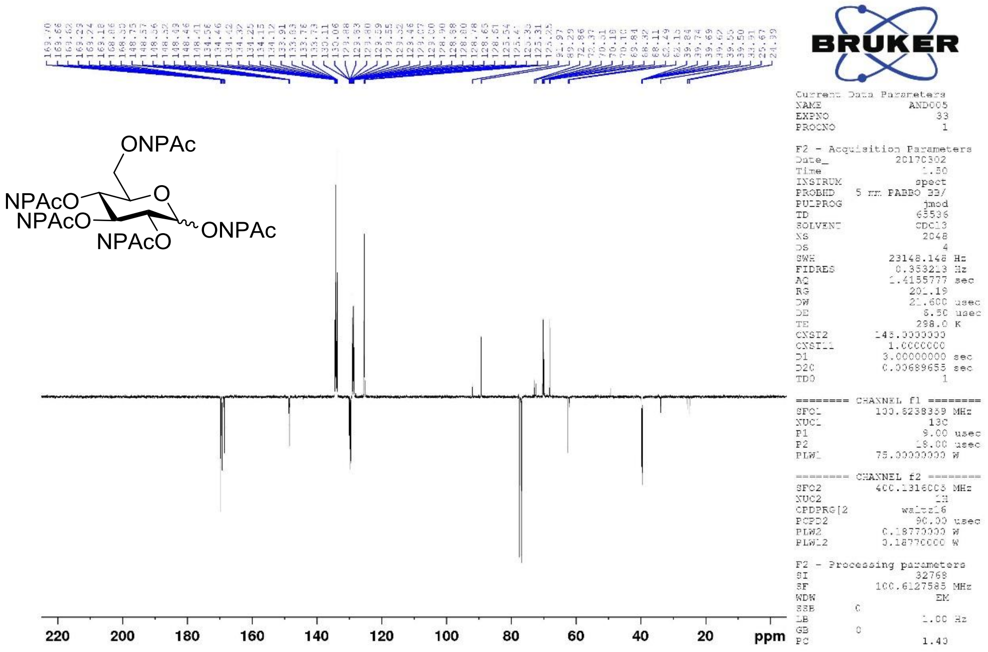

b. 1-Bromo-1-deoxy-2,3,4,6-tetra-*O*-(2-nitrophenyl)acetyl-α,D-glucopyranose (4)

^{1}H NMR (*d*-$CDCl_3$, 600 MHz)

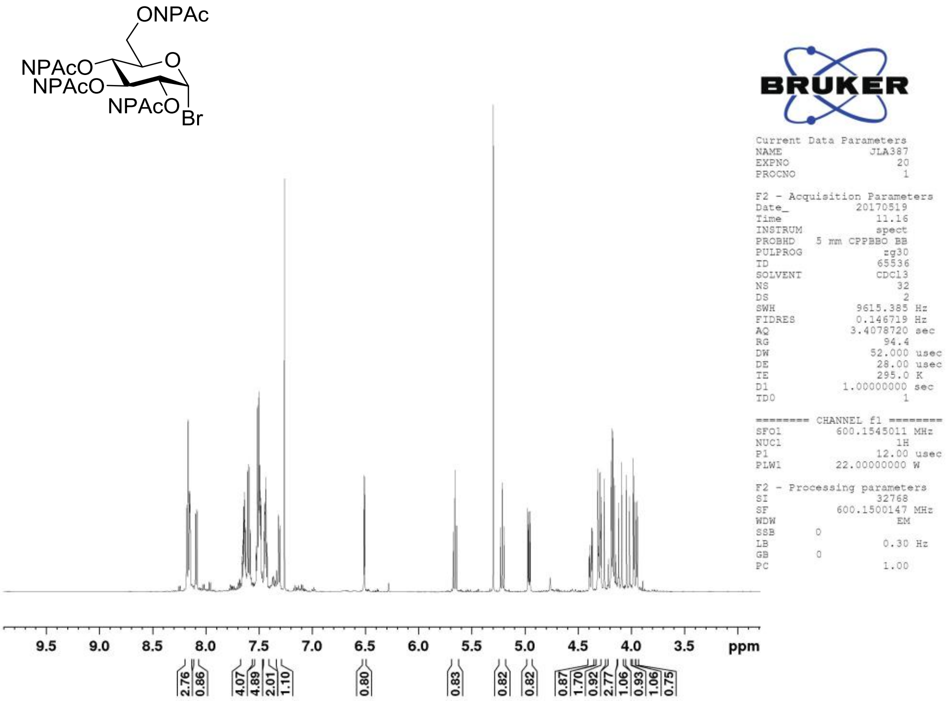

^{13}C NMR (*d*-$CDCl_3$, 150 MHz)

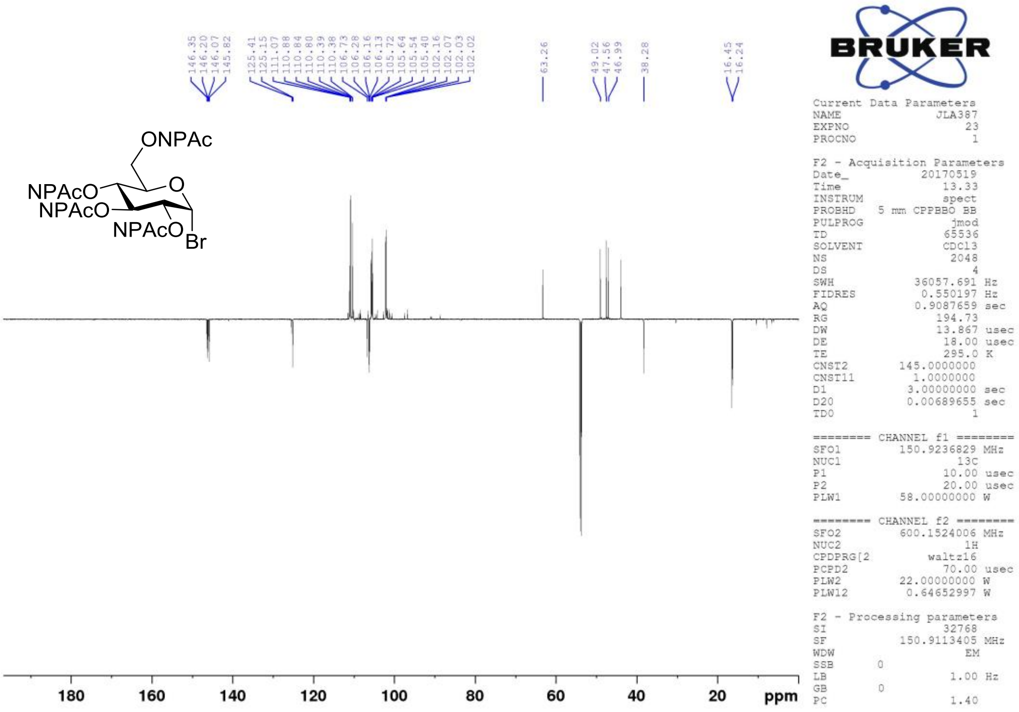

c. 2,3,4,6-Tetra-*O*-(2-nitrophenyl)acetyl-D-glucopyranose (5)

^{1}H NMR (*d*-$CDCl_3$, 400 MHz)

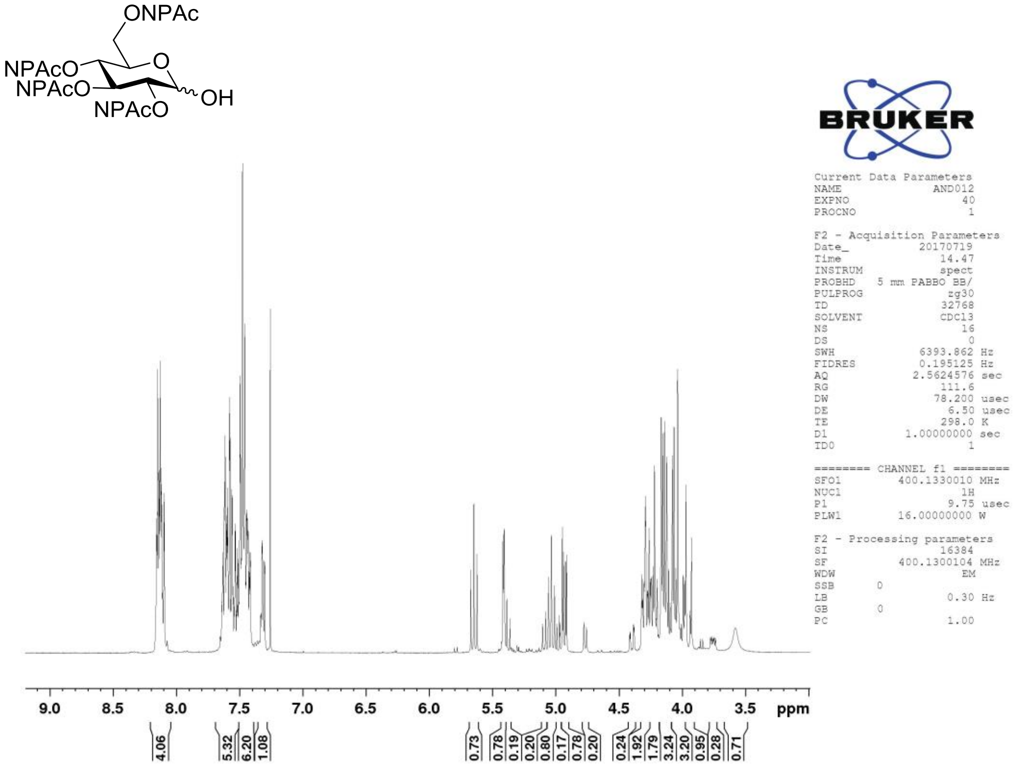

^{13}C NMR (*d*-$CDCl_3$, 100 MHz)

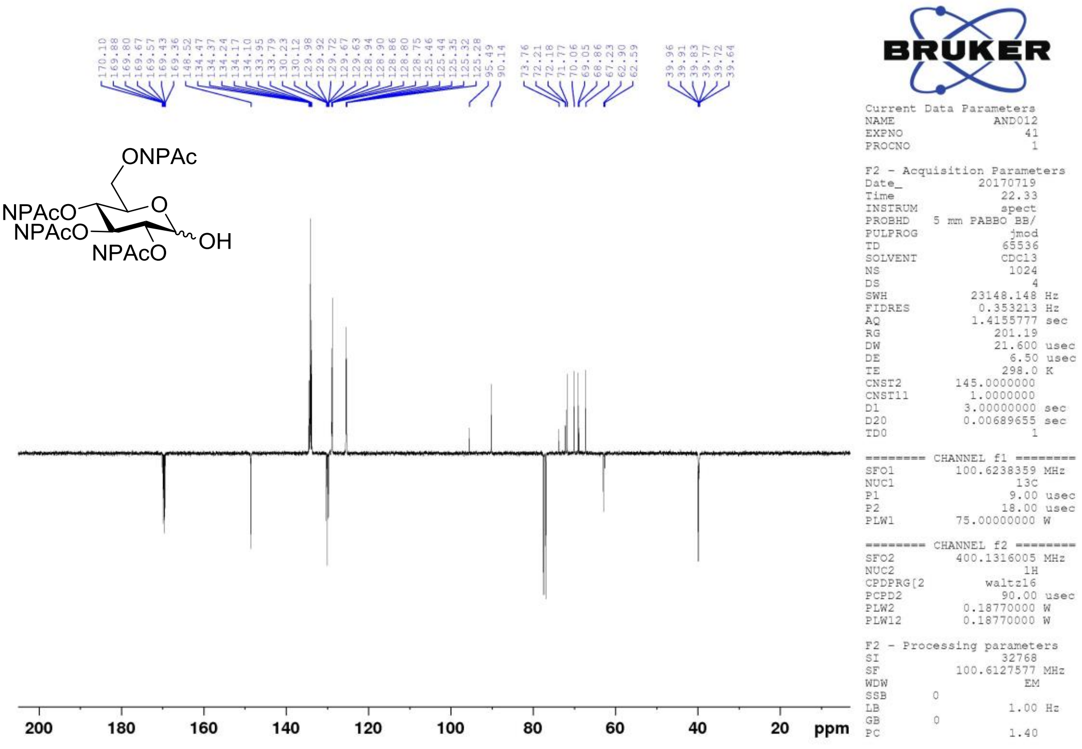

d. 2,3,4,6-Tetra-*O*-(2-nitrophenyl)acetyl-α,D-glucopyranosyl trichloroacetimidate (6)

^{1}H NMR (*d*-$CDCl_3$, 600 MHz)

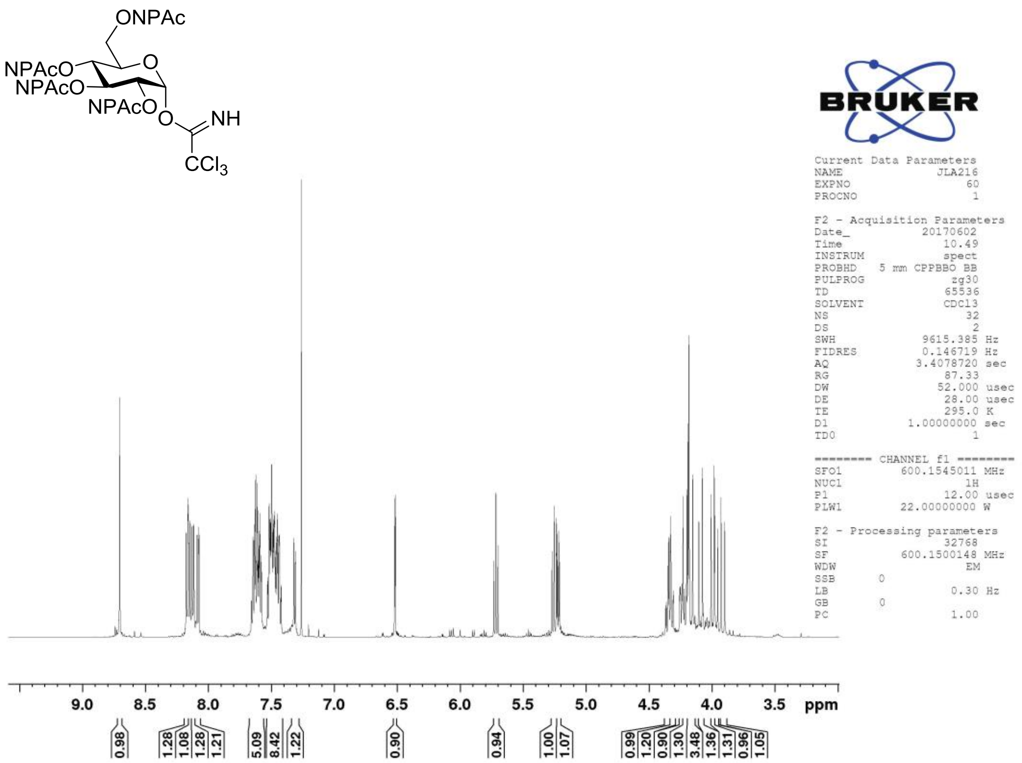

^{13}C NMR (*d*- $CDCl_3$, 150 MHz)

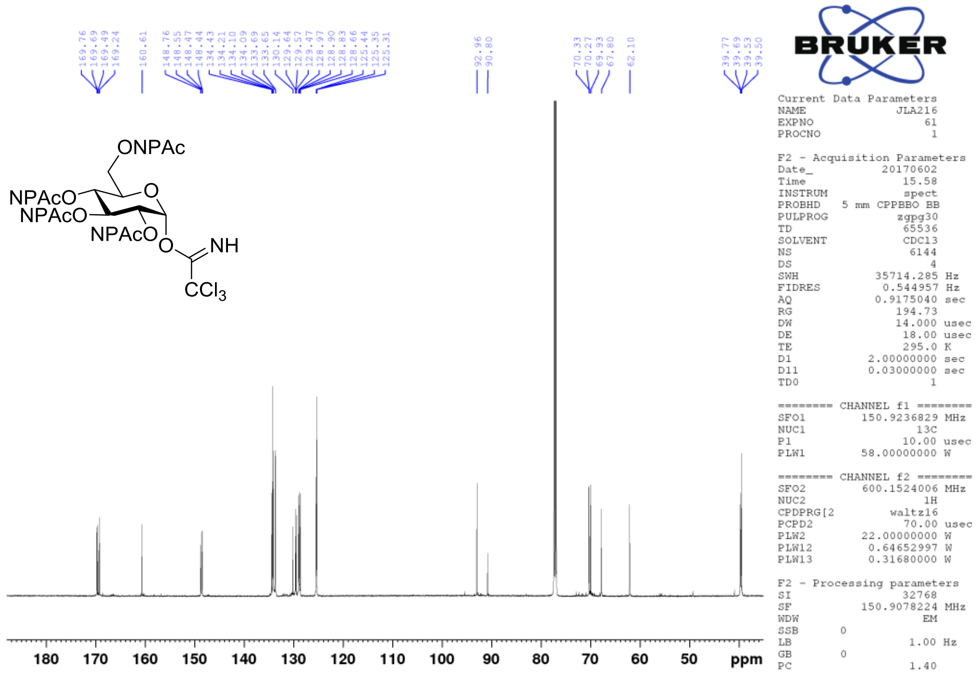

e. 1-Methylcyclohexyl-2,3,4,6-tetra-*O*-(2-nitrophenyl)acetyl-β,D-glucopyranoside (7)

^{1}H NMR (*d*- $CDCl_3$, 400 MHz)

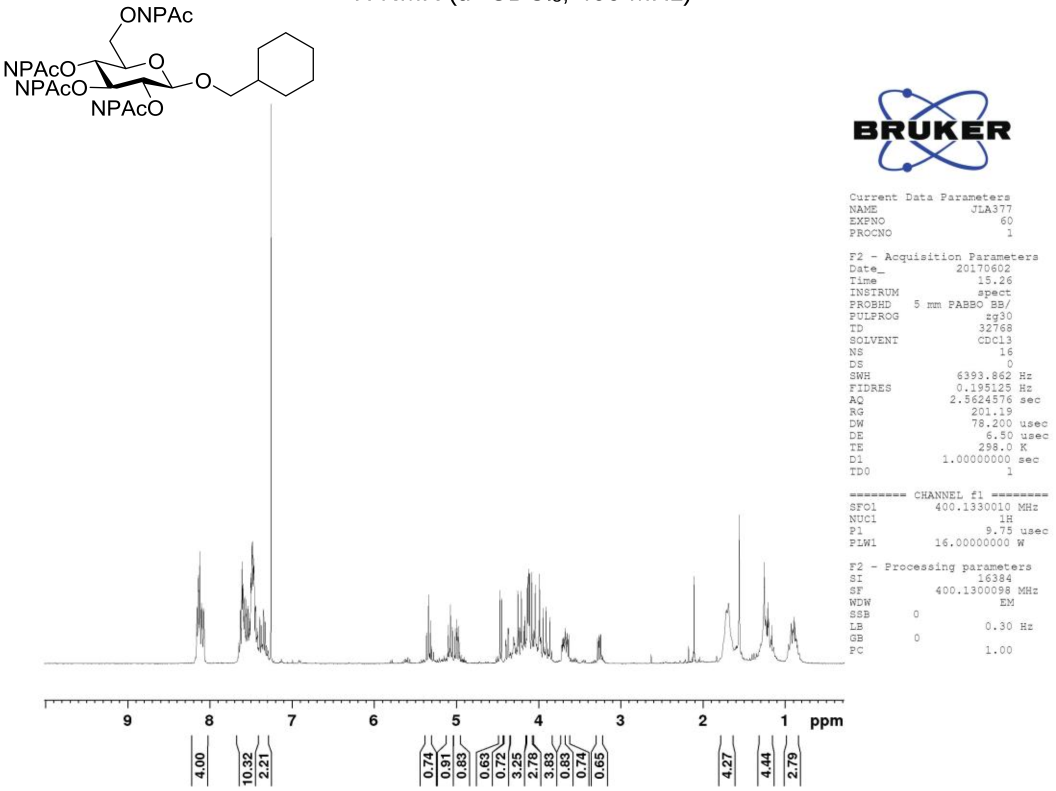

^{13}C NMR (*d*- $CDCl_3$, 100 MHz)

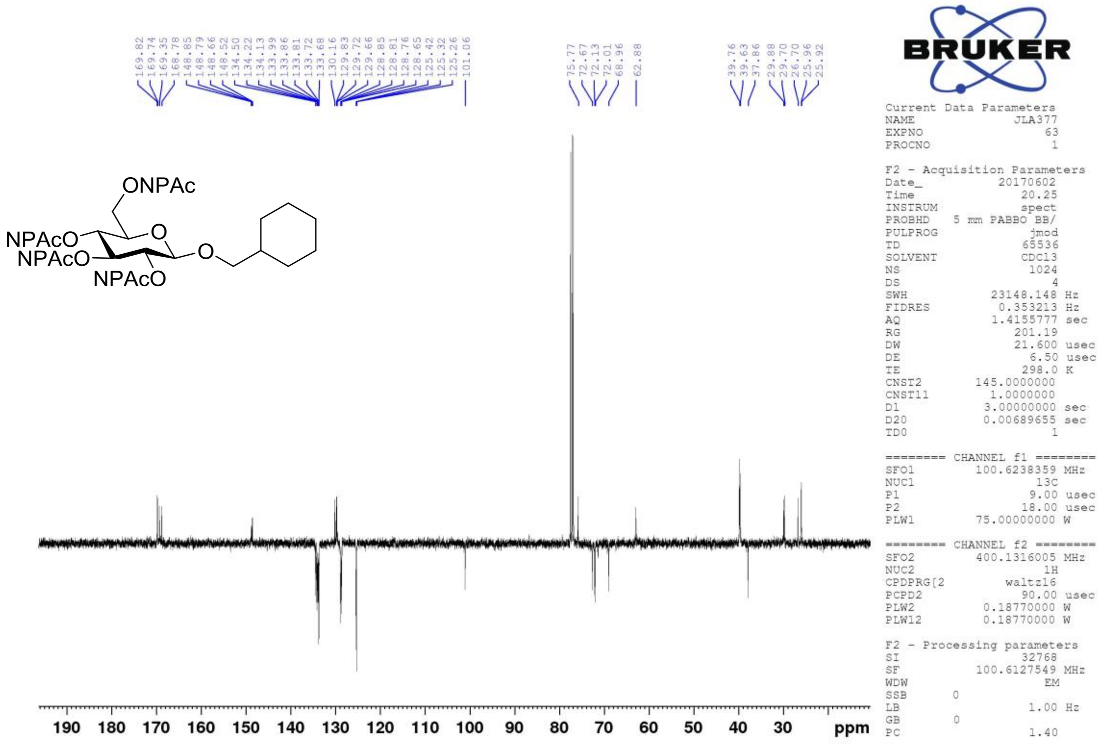

f. 1-(+)-Menthyl-2,3,4,6-tetra-*O*-(2-nitrophenyl)acetyl-β,D-glucopyranoside (8)

^{1}H NMR (*d*- CDCl$_3$, 400 MHz)

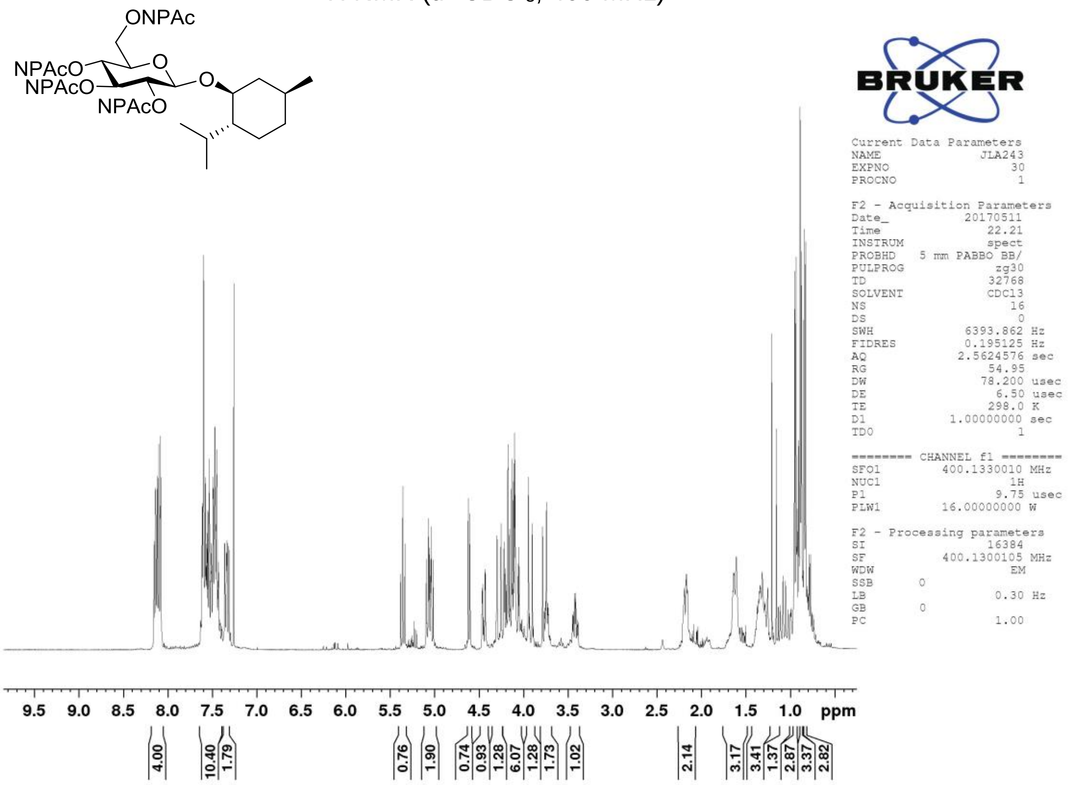

^{13}C NMR (*d*- CDCl$_3$, 100 MHz)

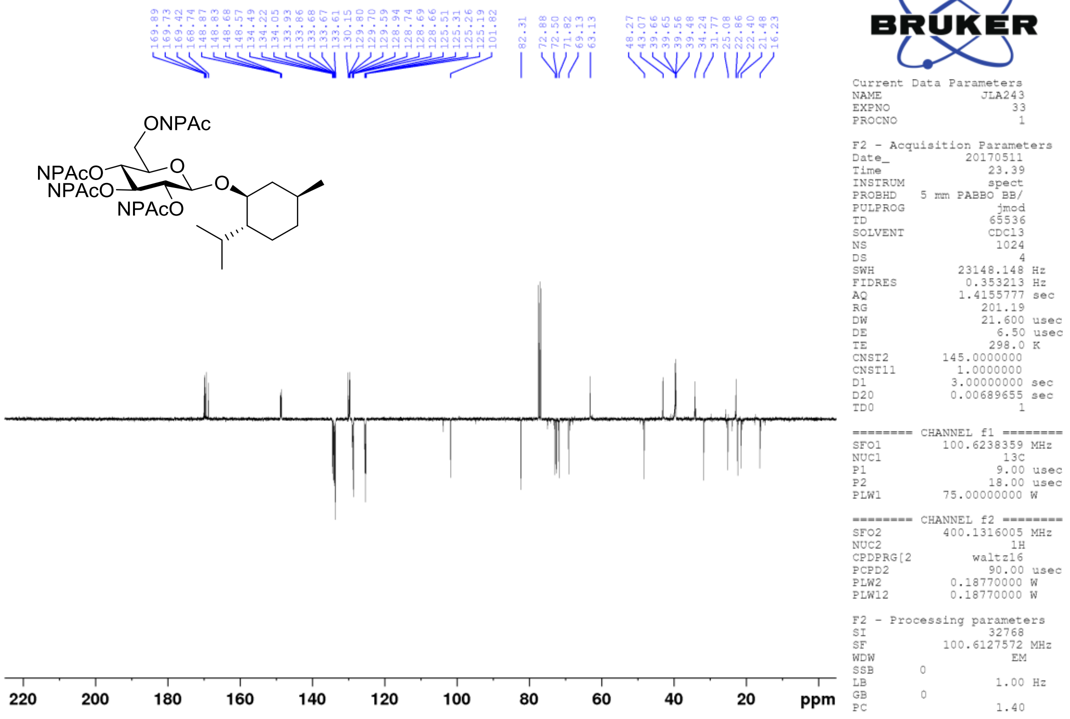

g. Methylcyclohexyl-β,D-glucopyranoside (9)

^{1}H NMR (*d*-MeOH, 400 MHz)

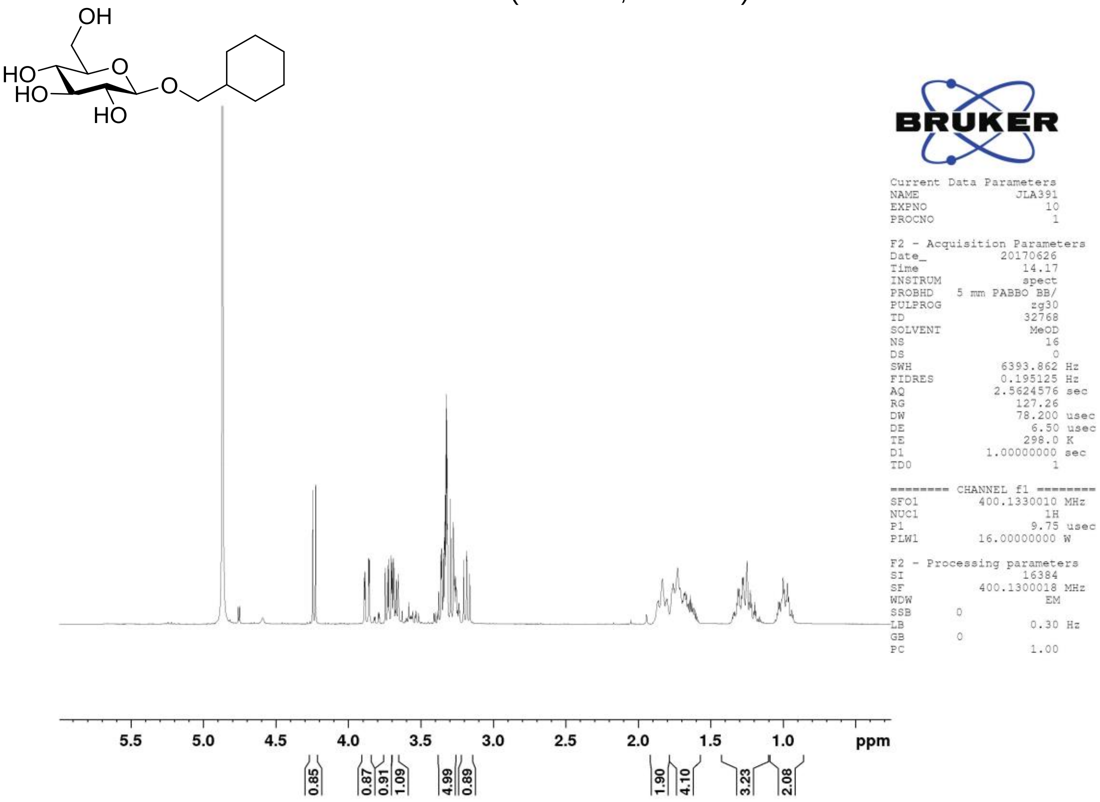

^{13}C NMR (*d*-MeOH, 100 MHz)

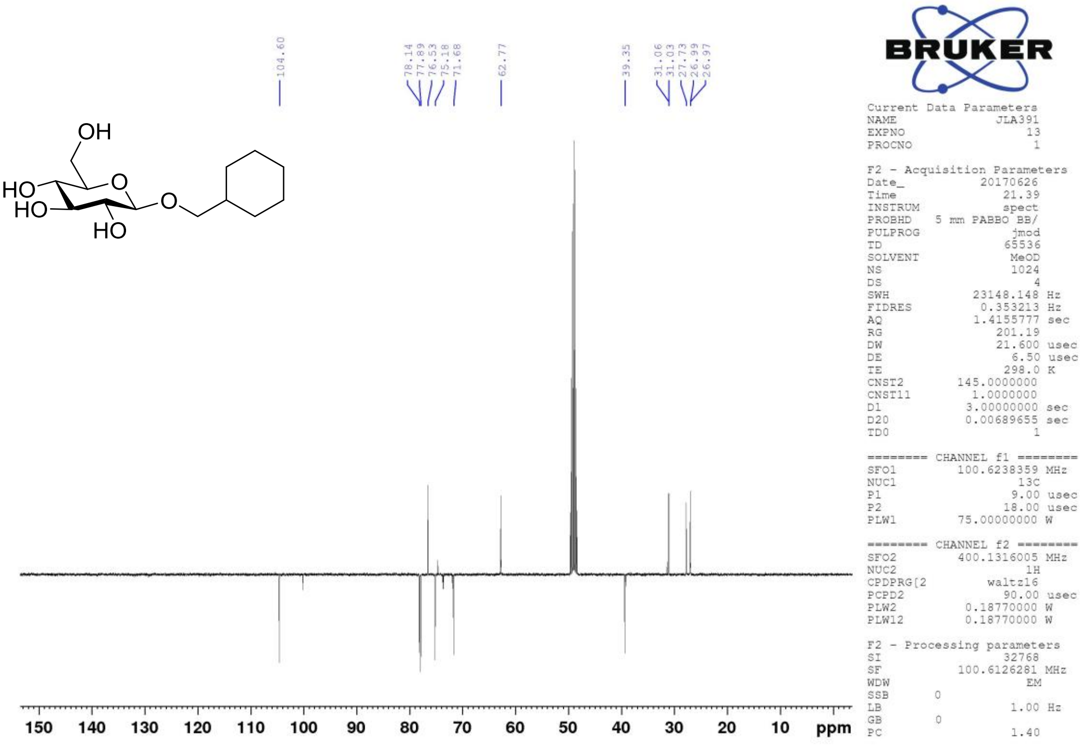

h. (+)-Menthyl-β,D-glucopyranoside (10)

^{1}H NMR (*d*-MeOH, 400 MHz)

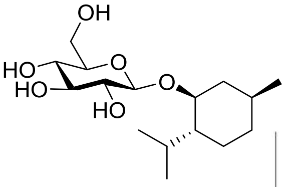

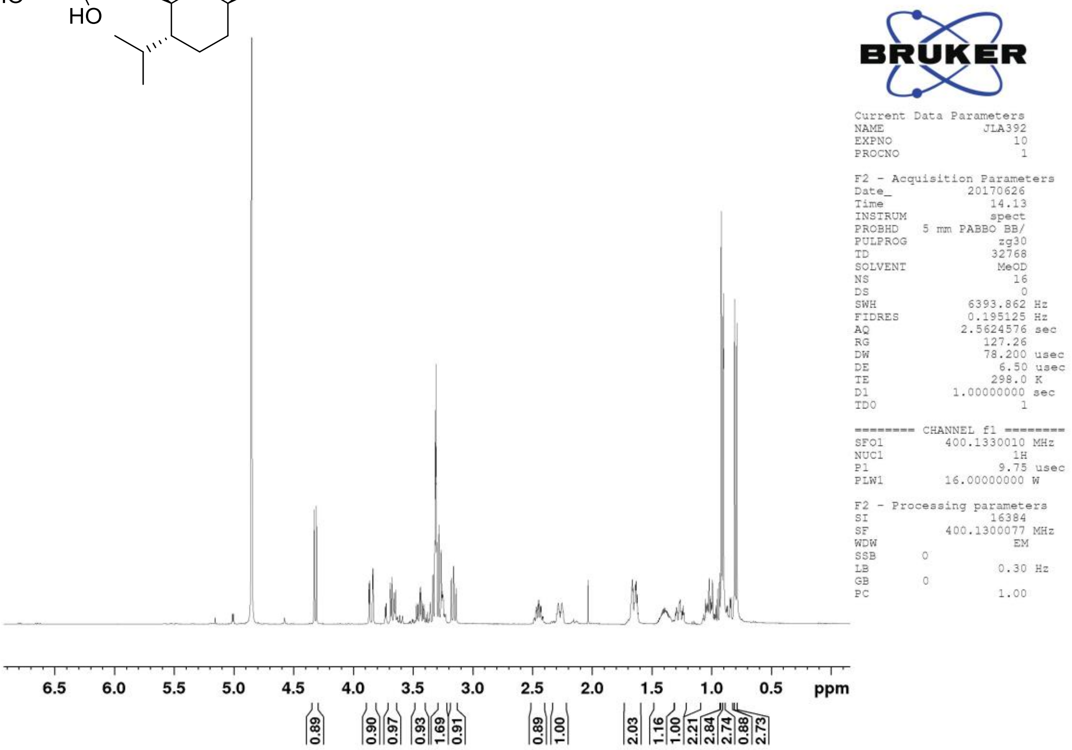

^{13}C NMR (*d*-MeOH, 100 MHz)

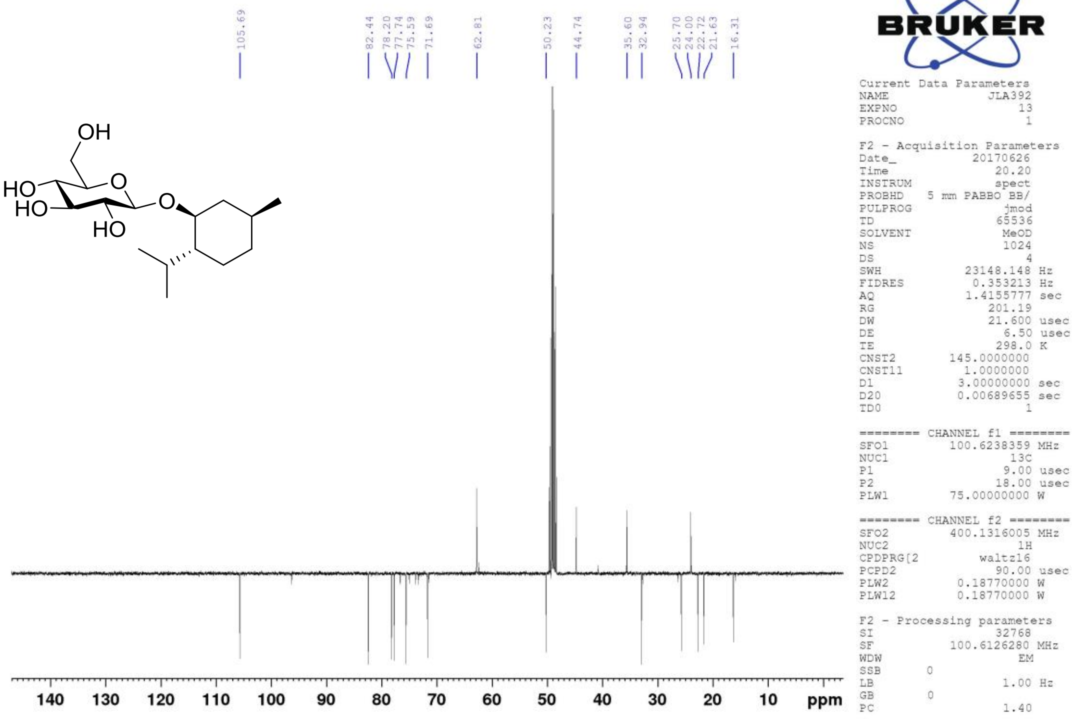

i. T2-*O*-β,D-glucoside (2)

^{1}H NMR (*d*-MeOH, 600 MHz)

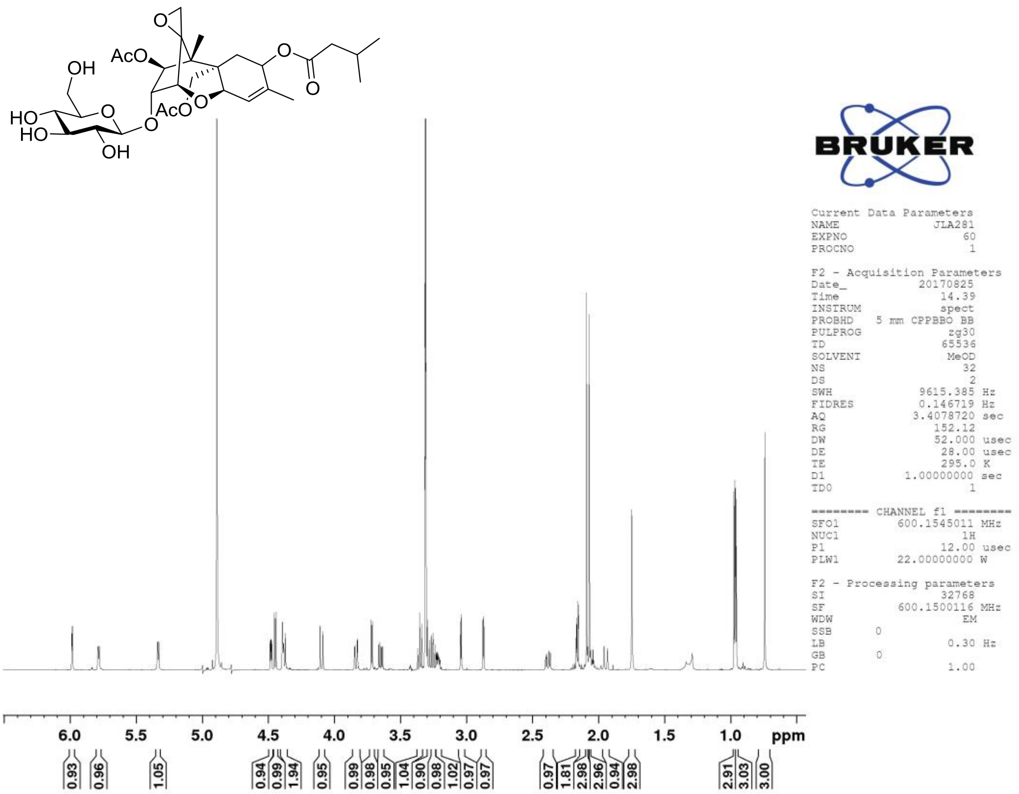

^{13}C NMR (*d*-MeOH, 150 MHz)

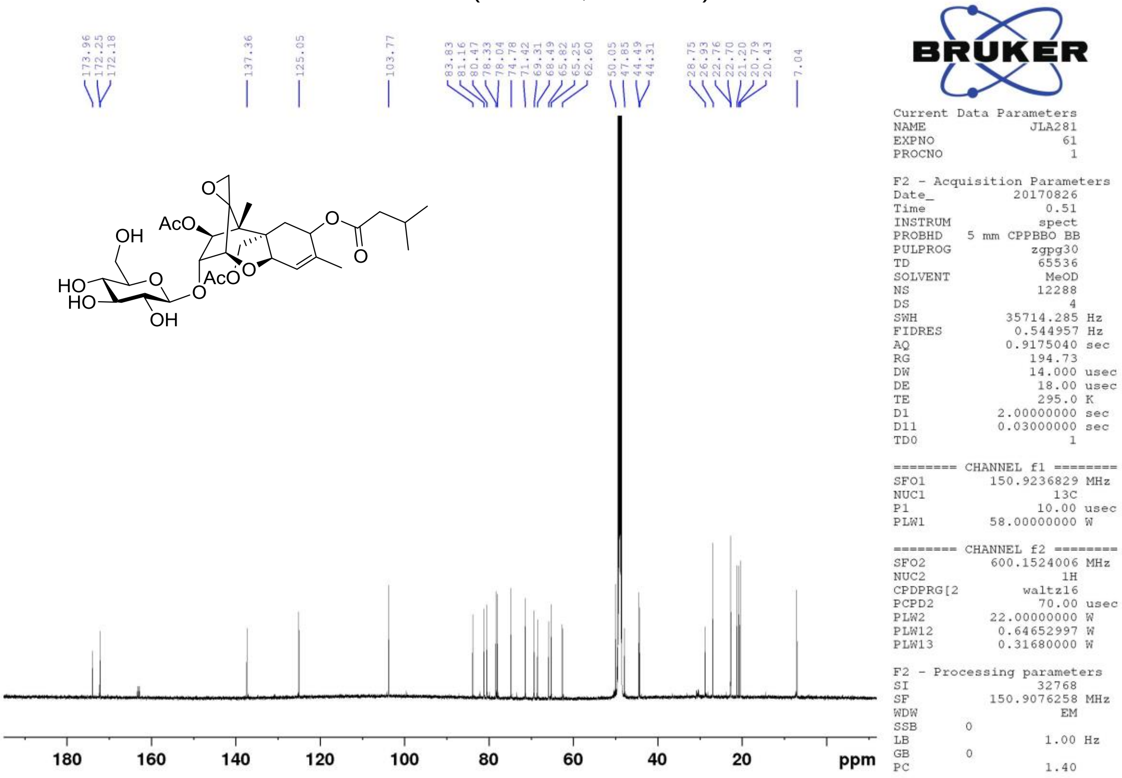

Supplement: Supplementary file 1 — Supporting Information [file EJOC-2018-2701-s001.pdf]
